# An optimized measles virus glycoprotein-pseudotyped lentiviral vector production system to promote efficient transduction of human primary B cells

**DOI:** 10.1016/j.xpro.2022.101228

**Published:** 2022-03-08

**Authors:** Eirini Vamva, Stosh Ozog, Els Verhoeyen, Richard G. James, David J. Rawlings, Bruce E. Torbett

**Affiliations:** 1 Department of Immunology and Microbiology, The Scripps Research Institute, La Jolla, CA, USA; 2 Center for Immunity and Immunotherapies, Seattle Children’s Research Institute, Seattle, WA, USA; 3 Department of Pediatrics, University of Washington School of Medicine, Seattle, WA, USA; 4 CIRI–International Center for Infectiology Research, Team EVIR, Université de Lyon, Lyon, France; 5 Institute for Stem Cell and Regenerative Medicine, Seattle, WA, USA

**Keywords:** Cell isolation, Flow Cytometry/Mass Cytometry, Immunology, Microbiology, Molecular Biology, Biotechnology and bioengineering

## Abstract

Measles virus envelope pseudotyped LV (MV-LV) can achieve high B cell transduction rates (up to 50%), but suffers from low titers. To overcome current limitations, we developed an optimized MV-LV production protocol that achieved consistent B cell transduction efficiency up to 75%. We detail this protocol along with analytical assays to assess the results of MV-LV mediated B cell transduction, including flow cytometry for B cell phenotypic characterization and measurement of transduction efficiency, and ddPCR for VCN analysis.

## Highlights

• We produced a robust measles virus pseudotyped lentiviral vector (MV-LV) protocol
• We show enhanced MV-LV mediated efficient transduction of primary human B cells
• We provide assays to quantify MV-LV titers, B cell transduction efficiency, and VCN
• Flow cytometry can be further used for primary B cell phenotypic characterization

## Graphical abstract

**Figure:**
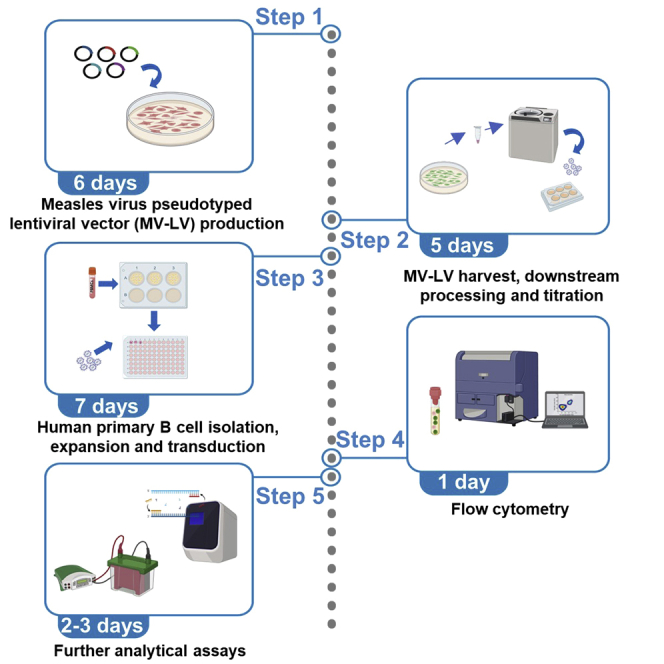

## Before you begin

This protocol describes the development of a Measles virus envelope gp pseudotyped (MV) lentiviral vector (MV-LV) production system which can be used to package complex transgene containing lentiviral vectors. We chose to evaluate the pMND-eGFP-eCD4-Ig lentiviral vector, a bicistronic vector that contains eGFP (Tsien, 1998) and the HIV-1 entry inhibitor immunoadhesin eCD4-Ig (Gardner et al., 2015), under the control of the constitutive MND (myeloproliferative sarcoma virus enhancer, negative control region deleted, dl587rev primer-binding site substituted) promoter (herein referred to as MND-eGFP-eCD4-Ig and described in key resources table). This transfer vector serves as a model for larger lentiviral bicistronic vectors which have been associated with reduced LV titers (Kumar et al., 2001; Morgan et al., 2019). We outline the steps to prepare high-titer MV-LV to transduce human primary B cells, a cell target important for gene therapy applications of therapeutic potential, including the treatment of genetic diseases (Hung et al., 2018), infectious diseases (Moffett et al., 2019; Nahmad et al., 2020), auto-immune disorders (Greiner et al., 2019), cancers (Page et al., 2020), and the possible use of chimeric B cell receptors (Pesch et al., 2019).

**Figure 1:**
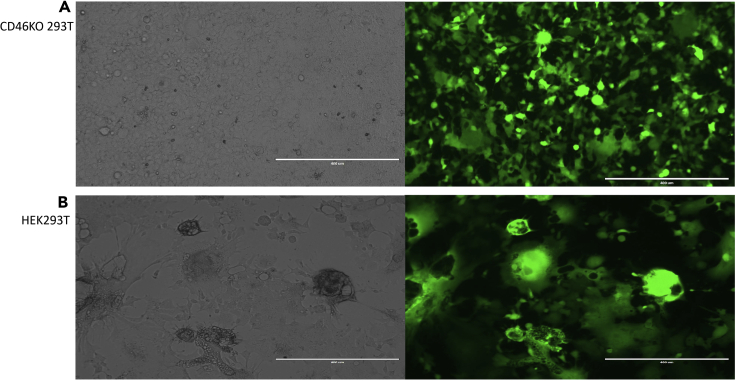
Transfection comparison between CD46KO 293T cells and HEK293T cells using all MV LV constructs (A) Representative 10× bright-field (left) and 488-nm 10× fluorescence (right) microscopy of transiently transfected CD46KO 293T cells. Picture was taken two days post transfection and indicates successful co-transfection of packaging plasmids to generate high titer MV-LV. (B) Representative 10× bright-field (left) and 488-nm 10× fluorescence (right) microscopy of transiently transfected HEK293T cells with the MV and packaging constructs two days post transfection, showing formation of major syncytia and observed cytotoxicity.

The eCD4-Ig gene was chosen for use given the expressed protein has antibody-like properties, suitable for B cell expression studies, and to ensure that MV-LVs encoding complex expression cassettes can also achieve high transduction efficiencies in human primary B cells. To improve MV-LV titers, we used the CD46KO 293T cell line (Ozog et al., 2019) which prevents cell membrane fusion when MV is expressed due to the loss of CD46 required for measles binding and entry and the resulting death of transfected producer cells (Figure 1). The use of the CD46KO 293T cell line also decreases co-purification of contaminating cell membranes in MV-LV supernatant which is consistent with higher primary B cell viability. We employed a small-scale transfection system with optimized plasmid ratios to ensure high transfection efficiency of the CD46KO 293T cell line, resulting in MV-LV which constantly transduced primary B cells with high efficiency. We have ensured appropriate purification and concentration of MV-LVs by introducing: i) a PES low protein binding filtering step for MV-LV purification and ii) a short high-speed ultracentrifugation step, for efficient MV-LV recovery and concentration.

VSV-G pseudotyped LVs are known to be inefficient at transducing primary human B cells (Serafini et al., 2004; Amirache et al., 2014). Alternative LV pseudotypes that can achieve higher B cell transduction rates suffer from low viral titers, making large-scale production and inclusion of long and complex transgenes challenging (Frecha et al., 2009; Lévy et al., 2012; Mock et al., 2012). Therefore, improvement of viral titers through a robust protocol that others in the field can easily reproduce is of paramount importance. Lastly, we present the workflow for successful isolation, expansion and transduction of primary B cells as well as the analytical assays required to assess transduction efficiency and viability of primary B cells.

### Preparing plasmids

**Timing: 3 days for plasmid preps, variable time to obtain the plasmids.**

1. Obtain the pCG-HΔ24, pCG-FΔ30, pMDLg/RRE and pRSV-Rev plasmids described herein, from Addgene or collaborating labs (see key resources table).

***Note:*** The transfer LV plasmid we used was MND-eGFP-eCD4-Ig. However, investigator’s transfer LVs will be specific for their applications. The system described here makes use of a 3^rd^ generation self-inactivating (SIN) lentiviral transfer vector containing the sequences spanning the CMV promoter, 5′LTR, a partial *gag* element, the RRE element, the constitutive MND promoter as an internal promoter, the eGFP-eCD4-Ig transgene, the WPRE element and the ΔU3-3′LTR.

2. Prepare sufficient quantities of each plasmid using the NucleoBond Xtra Midi Plus kit for transfection-grade plasmid DNA following the protocol provided below. Do not further dilute plasmids, use them at the highest concentration possible. Preferably around 2 μg/μL.

**CRITICAL:** The LV-MV packaging and transfer plasmids should be propagated using the One Shot™ Stbl3™ *E. coli* strain, developed to improve yields of plasmids that contain repetitive sequences.

### Culturing of CD46KO 293T lentiviral packaging cell line

**Timing: About 10 days**

3. CD46KO 293T packaging cells are cultured in complete DMEM (cDMEM, see materials and equipment) and are maintained at 37°C in a humidified atmosphere containing 5% CO_2_. Passage cells for 10 days post thawing and prior to the transfection protocol to ensure that cells are metabolically active, thus enabling efficient co-transfection of the 5 required plasmids. CD46KO 293T cells have been passaged up to 2 months without an observed decrease in the produced MV-LV titers. Subculture cells every 5 days using a 1:10 split ratio in sterile conditions.

***Note:*** CD46KO 293T are weakly adherent and detach easily from the culture dish, therefore it is important to coat the transfection plates with poly L-Lysine solution before seeding (see: MV pseudotyped lentiviral vector production).

***Note:*** We have determined that concentrated MV-LV supernatant from a single 1 × 10 cm^2^ transfection plate is required to efficiently transduce 3 × 10^5^ human primary B cells seeded in 1 well of a 96 well-plate. For B cell studies, technical triplicates are suggested per experimental condition. As a general rule, seed 3× the number of 1 × 10 cm^2^ transfection plates per condition of interest to produce sufficient MV-LV. To ensure the availability of adequate numbers of CD46KO 293T cells for MV-LV production determine in advance the number of transfection plates and therefore the number of CD46KO 293T cells required.

### Culturing of Nalm6 cells

**Timing: 1 week**

4. The Nalm6 B cell precursor leukemia cell line is grown in complete RPMI (cRPMI, see materials and equipment) and maintained at 37°C in a humidified atmosphere containing 5% CO_2_. Nalm6 cells grow as floating clusters of cells and can be collected by directly pipetting the cells from the tissue culture plate. We prefer the use of 10 cm^2^ plates for the passaging of Nalm6 cells. Passage Nalm6 cells up to 1 month before needing to start new cultures.

### Culturing of primary B cells

**Timing: 2 weeks**

5. Obtain ethically sourced isolated peripheral blood human mononuclear cells (PBMCs). The PBMCs used in this study were obtained from the Specimen Processing and Research Cell Bank at the Fred Hutchinson Cancer Research Center under approved Human Subject Protocol. Isolated B cells (see primary B cell isolation and activation) can be activated and expanded in B cell expansion media (see materials and equipment) for up to two weeks. Transductions are performed 2 days post isolation and activation.

***Note:*** The optimal concentration of human primary B cells is 1.5 × 10^6^ cells/mL (in any culture condition). The following suggested protocol achieves 5–6× expansion of cells in B cell expansion media (see: Materials and equipment). Transduced B cells can be up to 10% less viable than mock transduced primary B cells. It is necessary to consider these findings when determining the number of B cells required for downstream experimental studies.

## Key resources table

**Table:** 

REAGENT or RESOURCE	SOURCE	IDENTIFIER
**Antibodies**		

Brilliant Violet 605™ anti-human CD19 antibody (dilution 1:200)	BioLegend	Cat#302243
Brilliant Violet 785™ anti-human CD3 antibody (dilution 1:200)	BioLegend	Cat#317329
PE/Cyanine7 anti-human IgM antibody (dilution 1:200)	BioLegend	Cat#314532

**Bacterial strains**		

One Shot™ Stbl3™ Chemically Competent *E. coli*	Invitrogen™	Cat#C737303

**Biological samples**		

PBMCs	Fred Hutchinson Cancer Research Center	Specimen Processing and Research Cell Bank

**Chemical, peptides, and recombinant proteins**		

DMEM, high glucose, GlutaMAX™ Supplement	Gibco	Cat#10566016
IMDM, no phenol red	Gibco	Cat#21056023
RPMI with Glutamine	Corning	Cat#10-040-CV
Hank’s Balanced Salt Solution, 1× without calcium, magnesium and phenol red (HBSS)	Corning	Cat#21-022-CV
EasySep™ Buffer	STEMCELL Technologies	Cat#20144
Fetal bovine serum (FBS)	Sigma-Aldrich	Cat#F0926-500ML
Beta-mercaptoethanol	Gibco	Cat#21985-023
TryplE	Thermo Fisher Scientific	Cat#12604013
Penicillin-Streptomycin (10,000 U/mL)	Gibco	Cat#15140122
polyL-Lysine solution	Sigma-Aldrich	Cat#P4707-50 mL
PBS	Cytiva	Cat#SH30256.01
Cytofix/Cytoperm™ Fixation and permeabilization solution	BD Biosciences	Cat#554722
Perm/Wash™ Buffer	BD Biosciences	Cat#554723
Cytoperm™ Permeabilization Buffer Plus	BD Biosciences	Cat#561651
AbC™ Total Antibody Compensation Bead Kit	Invitrogen	Cat#A10513

**Chemicals**		

Polybrene	Millipore Sigma	Cat#TR-1003-G
Sucrose	Fisher BioReagents	Cat#BP220-1
Ampicillin	Sigma-Aldrich	Cat#A5354
MCD40L (100 ng/mL)	Enzo Life Sciences	Cat#ALX-522-110-C010
CpG ODN 2006 (1 μg/m)l	InvivoGen	Cat#NC9902842/tlrl-2006-1
IL-2 (50 ng/mL)	PeproTech	Cat#200-02
IL-10 (50 ng/mL)	PeproTech	Cat#200-10
IL-15 (10 ng/mL)	Miltenyi Biotec	Cat#130-095-765
Miller’s LB	Corning	Cat#46-050-CM
AF350 LIVE/DEAD^TM^ fixable violet dead cell stain (dilution 1:1000)	Thermo Fisher Scientific	Cat#L34964

**Critical Commercial kits**		

NucleoBond Xtra Midi Plus kit for transfection-grade plasmid DNA	MACHEREY-NAGEL	Cat#REF 740412.50
Profection® Mammalian transfection system	Promega	Cat#E1200
EasySep Human B cell Isolation Kit	STEMCELL Technologies	Cat#17954
The "Big Easy" EasySep™ Magnet	STEMCELL Technologies	Cat#18001
DNeasy Blood and Tissue kit	QIAGEN	Cat#69504
Lenti-X p24 Rapid Titer Kit	TaKaRa Bio	Cat#632200

**Experimental models: Cell lines**		

CD46KO 293T Cells	Torbett lab ∗The Scripps Research Institute, La Jolla, CA, USA, is in the process of depositing the cell line at the National Gene Vector Biorepository (https://www.ngvbcc.org/Home.action)	Ozog et al. (2019)
Nalm6 cells	Torbett lab	https://www.atcc.org/products/crl-3273

**Oligonucleotides**		

HIV-1 Fw primer	This paper	5′-CTGTTGTGTGACTCTGGTAACT-3′
HIV-1 Rv primer	This paper	5′-TTCGCTTTCAAGTCCCTGTT-3′
MKL2 Fw primer	This paper	5′-AGATCAGAAGGGTGAGAAGAATG-3′
MKL2 Rv primer	This paper	5′-GGATGGTCTGGTAGTTGTAGTG-3′
HIV-1 probe	This paper	5′-FAM/AAATCTCTA/ZEN/GCAGTGGCGCCCG/3IABkFQ-3′
MKL2 probe	This paper	5′-HEX/TGTTCCTGC/ZEN/AACTGCAGATCCTGA/3IABkFQ-3′

**Recombinant DNA**		

Measles virus Edmonston strain hemagglutinin (pCG-HΔ24)	Ozog et al. (2019)	Genbank ID AB583749.1
Measles virus Edmonston strain fusion (pCG-FΔ30)	Ozog et al. (2019)	Genbank ID U03657.1
pMDLg/RRE	(Dull et al., 1998)	Addgene; #12251
pRSV-Rev	(Dull et al., 1998)	Addgene; #12253
p-MND-eGFP-eCD4-Ig	Torbett lab	eCD4-Ig Gardner et al., 2015

**Software and algorithms**		

Calculator for determining the number of copies of a template	Andrew Staroscik	http://cels.uri.edu/gsc/cndna.htmL
FlowJo 10.7.1 software	FlowJo (Becton Dickinson)	https://www.flowjo.com/
GraphPad Prism 9.3.0	GraphPad	https://www.graphpad.com/scientific-software/prism/
BioRender	BioRender	https://biorender.com/
Quantasoft software	Bio-Rad	Cat#186-3007

**Other**		

QX100 ddPCR	Bio-Rad	Cat#186-3001
QX droplet generator	Bio-Rad	Cat#186-3002
QX100 droplet reader	Bio-Rad	Cat#186-3003
Droplet reader oil	Bio-Rad	Cat#186-3004
Droplet generation oil	Bio-Rad	Cat#186-3005
PX1 PCR plate sealer	Bio-Rad	Cat#181-4000
ddPCR Supermix for probes	Bio-Rad	Cat#186-3010
DG8 cartridges	Bio-Rad	Cat#186-4008
DG8 gaskets	Bio-Rad	Cat#186-3009
Droplet generator cartridge holders	Bio-Rad	Cat#186-3051
96 well plates semi-skirted	Bio-Rad	Cat#HSL9601
0.22 μm PES filter	Millipore	Cat#SLGPR33RS
14 mL polystyrene round bottom tube	Falcon	Cat#352057
Ultracentrifuge tubes	Nalgene	Cat#3110-0500
Bio-Rad T100 Thermal Cycler	Bio-Rad	Cat#1861096
Optima^TM^ L-90K Ultracentrifuge	Beckman Coulter	Cat#SKU: 8043-30-1191
SW28 Ti rotor	Beckman Coulter	Cat#342204
BD LSRII	BD Biosciences	N/A
EVOS M5000 Imaging system	Thermo Fisher Scientific	Cat#AMF5000

## Materials and equipment

**Table:** AmpLB

Reagent	Final concentration	Amount
Miller’s LB	1×	1 L
Ampicillin	50 μg/mL	1,000 μL

Store at 4°C for up to 1 month.

**Table:** cDMEM

Reagent	Final concentration	Amount
DMEM, high glucose, GlutaMAX™ Supplement	1×	500 mL
FBS	10%	50 mL
Penicillin-Streptomycin	100 U/mL	5 mL

Store at 4°C for up to 1 month.

**Table:** 3%FBS-DMEM

Reagent	Final concentration	Amount
DMEM, high glucose, GlutaMAX™ Supplement	1×	500 mL
FBS	3%	17 mL

Store at 4°C for up to 1 month.

**Table:** cRPMI

Reagent	Final concentration	Amount
RPMI with glutamine	1×	500 mL
FBS	10%	50 mL
Penicillin-Streptomycin	100 U/mL	5 mL

Store at 4°C for up to 1 month.

**Table:** B cell basic media

Reagent	Final concentration	Amount
IMDM	1×	500 mL
FBS	10%	50 mL
Beta-mercaptoethanol	1× (55 μM in PBS)	500 μL

Store at 4°C for up to 1 month.

**Table:** B cell expansion media

Reagent	Final concentration	Amount
B cell basic media	1×	10 mL
MCD40L	100 ng/mL	10 μL
CpG ODN 2006	1 μg/mL	10 μL
IL-2	50 ng/mL	10 μL
IL-10	50 ng/mL	10 μL
IL-15	10 ng/mL	10 μL

Store at 4°C for up to 1 week.

***Note:*** Given the instability of the diluted additives included in the B cell expansion media, make sure to prepare only the volume required for the experimental procedure just before use. 1× B cell expansion medium can be stored in 4°C up to one week.

**Table:** 

Oligonucleotide	Sequence
HIV-1 Fw primer	5′-CTGTTGTGTGACTCTGGTAACT-3′
HIV-1 Rv primer	5′-TTCGCTTTCAAGTCCCTGTT-3′
MKL2 Fw primer	5′-AGATCAGAAGGGTGAGAAGAATG-3′
MKL2 Rv primer	5′-GGATGGTCTGGTAGTTGTAGTG-3′
HIV-1 probe	5′-FAM/AAATCTCTA/ZEN/GCAGTGGCGCCCG/3IABkFQ-3′
MKL2 probe	5′-HEX/TGTTCCTGC/ZEN/AACTGCAGATCCTGA/3IABkFQ-3′

***Alternatives:*** Alternative centrifuges and ultracentrifuges capable of the listed performance are acceptable. Alternative thermal cyclers capable of reaching the listed temperatures are acceptable. Other flow cytometers equipped with the same lasers and capable of multicolor analysis are appropriate alternatives for the BD LSRII. Traditional qPCR could be performed using the same sets of primers/probes as an alternative to ddPCR, but we cannot guarantee the same level of accuracy.

## Step-by-step method details

### Plasmid preparation

**Timing: 3 days**

3^rd^ generation lentiviral vectors are made of 4 independent plasmids coding for the transfer vector, the packaging constructs and the envelope. The following steps ensure transfection-grade production of plasmid constructs.

1. Transform different vials of One Shot™ Stbl3™ Chemically Competent *E. coli* cells with 10 ng of pCG-HΔ24, pCG-FΔ30, pMDLg/RRE, pRSV-Rev, p-MND-eGFP-eCD4-Ig (or the transfer vector of interest) following the manufacturer’s protocol (https://www.thermofisher.com/document-connect/document-connect.html?url=https%3A%2F%2Fassets.thermofisher.com%2FTFS-Assets%2FLSG%2Fmanuals%2Foneshot_stbl3_man.pdf).

***Note:*** This *E. coli* strain is recommended for use when working with sequences containing direct repeats, including lentiviral transfer vector DNA.

2. 16 h later, pick up a single colony from each transformation plate and inoculate into a 250 mL ampLB medium (see materials and equipment) in a 1 L flask. Cover the top of the flask with foil and create small holes with a 20 or 200 uL pipet tip. Incubate the flask at 37°C with constant shaking (300 rpm) for 16 h overnight.

**CRITICAL:** The concentration and quality of plasmid DNA are very critical parameters to achieve high transfection efficiencies, especially in a protocol that requires the co-transfection of 5 independent plasmids. Yield and quality of plasmid DNA depend, amongst other parameters, on the bacterial host strain and on the growth conditions. Low bacterial growth will inevitably lead to low plasmid yield, while culture overgrowing can lead to a higher percentage of dead or starving cells, resulting in potentially degraded or contaminated plasmid DNA. Create holes on the surface of each flask and use 1L flasks (four times the volume of the culture) to maintain LB saturated with oxygen.

3. 16 h later collect and load the culture in a centrifuge tube. Spin at 4,000×*g* for 30 min at 4°C.
4. Resuspend the bacterial pellet in 12 mL of Resuspension Buffer RES+RNase A by pipetting the cells up and down and vortex to ensure sample homogeneity.
5. Add 12 mL of lysis buffer, LYS (ensure that lysis buffer is not precipitated), to the bacterial lysate and mix by gently inverting the tube 5 times. Incubate at room temperature (RT=22°C–24°C) for 5 min.
  **CRITICAL:** Ensure gentle mixing of the culture to prevent membrane shear that can result to bacterial chromosomal DNA release contaminating the plasmid DNA suspension.
6. Meanwhile apply 12 mL of equilibration buffer EQU onto the rim of the NucleoBond® Xtra Column Filter to ensure high yield.
7. Neutralize the lysis buffer by adding 12 mL of buffer NEU. Mix thoroughly until the lysate is colorless. Load lysate on the NucleoBond® Xtra Column Filter.
8. Add 10 mL of Wash Buffer EQU. Once this step is complete, discard the Column Filter and perform another wash step by adding 12 mL of EQU buffer.
9. Place the column in a sterile 50 mL falcon tube. Elute plasmid DNA with 5 mL of pre-warmed Elution buffer ELU.
10. Precipitate eluted plasmid DNA with 3.5 mL of isopropanol and thorough mixing. Centrifuge at 4,500 × *g* for 1 h. Alternatively, add the isopropanol to the elution column, to simultaneously increase the yield of plasmid DNA.
11. Decant supernatant and add 10 mL of 70% ethanol. Wash the pellet by resuspending it in 70% ethanol and ultracentrifuge as above. Remove supernatant and allow pellet to air dry at RT for 10 min.

**CRITICAL:** Resuspend the pellet in ethanol and run an hour long precipitation step to increase the quality for transfection grade plasmid DNA.

12. Dissolve DNA pellet in 500 μL of sterile water. Quantify and qualify plasmid DNA with UV spectrometry and evaluate by restriction digests, respectively. Aliquot plasmid DNA to avoid multiple rounds of freeze-thawing and store in −20°C up to 2 years.

**CRITICAL:** Ensure the quality of plasmid DNA by measuring the 260/230 ratio. Generally, a ratio close to 2.0 is considered pure. Following this protocol the expected yield is ≥2 μg/mL. High plasmid concentrations and plasmid supercoiling are important parameters to achieve high transfection efficiency. Do not proceed if your concentration is less than ∼1.5 μg/mL.

### MV pseudotyped lentiviral vector production

**Timing: 4 days**

The following section details the steps required to produce high titer MV-LV, including transfection of CD46KO 293T cells with the use of plasmids generated as described above as well as viral vector supernatant collection and purification.

Day 1

13. Prepare 10 cm^2^ tissue culture plates with sterile 0.01% poly-L-lysine solution using a laminar flow tissue culture hood to ensure a sterile working space.
  a. Plates are usually prepared by pipetting ∼2 mL of solution into one plate, then tipping and swirling until a visible thin layer covers the entire bottom of the plate.
  b. The remaining solution is then pipetted and added to the next plate and the process is repeated as before.
  c. Aspirate any remaining solution from plates and let them completely dry (∼2 h) inside the tissue culture hood.
  d. Proceed to seeding CD46KO 293T cells without washing off the poly-L-lysine coated plates.

***Note:*** The transfection conditions described below refer to a single 10 cm^2^ plate to ensure high transfection efficiency; a major challenge in producing high titer MV-LV. Attempts to increase scalability of MV-LV production led to decreased transfection efficiency and therefore lower viral titers. Given the problems of scalability, perform plasmid transfections in single 10 cm^2^ plates and then proceed to collating and mixing the viral supernatant from 3 independent 10 cm^2^ transfection plates for purification and subsequent use for transduction of human primary B cells. This will allow sufficient MV-LV supernatant to be produced for the successful transduction of 3 technical replicates for each B cell experimental condition of interest.

14. 2 h later, split and count CD46KO 293T cells. CD46KO 293T cells were split using the TrypLE dissociating enzyme. TrypLE was preferred over other dissociating agents due to its recombinant, animal origin-free nature. Seed 5 × 10^6^ cells per poly-L-lysine coated plate in 10 mL of cDMEM. Incubate cells for 16 h in a 37°C, humidified 5% CO_2_ incubator.

Day 2

15. 16 h later, remove cDMEM medium from the CD46KO 293T cell containing plates and replace with 10 mL of fresh cDMEM. Meanwhile allow the reagents from the Profection® Mammalian transfection system to thaw in RT for approximately 2 h.

**CRITICAL:** CD46KO 293T cells need to be 90% confluent on the day of plasmid transfection. Lower confluency is associated with lower MV-LV viral titers. Higher confluency will lead to a higher cell death rate which will also result in reduced viral titers in addition to reduced prep purity.

16. Calculate the volume of each plasmid required per transfection reaction per 10 cm^2^ plate of CD46KO 293T cells, as shown below. Plasmid DNA mass was calculated using the following dsDNA copy number calculator formula: number of copies = (amount ∗ 6.022 × 10^23^) / (length ∗ 1 × 10^9^ ∗ 650) (http://cels.uri.edu/gsc/cndna.htmL).

**Table:** 

Plasmid	μg per 10 cm^2^ transfection plate	Plasmid length (bp)	Copy no
pMDLg/RRE	5	8890	5.21 × 10^11^
pRSV-Rev	2.5	4180	5.54 × 10^11^
pCG-HΔ24	1.8	6386	2.61 × 10^11^
pCG-FΔ30	1.8	6192	2.69 × 10^11^
pMND-eGFP-eCD4-Ig	15	10632	1.31 × 10^12^

17. Add the required plasmid DNA mass in a 1.5 mL Eppendorf tube. Add 62 μL of 2.5 M CaCl_2_ per transfection to the DNA mixture and pipette up and down gently until mixed. Add sterile water to a total volume of 500 μL. Vortex mix and briefly spin the contents of the tube.
18. Add 500 μL of 2× Hepes Buffer Solution (HBS) pH 7.0 (reagent is part of the Profection® Mammalian transfection system kit that is listed in the key resources table) in a 15 mL polystyrene conical tube.

**CRITICAL:** The reaction is pH dependent. Ensure that the pH of the Hepes Buffer is 7.0. Any deviation from this suggested pH value will negatively affect transfection efficiency and functional titers.

19. Bubble filtered (sterile) air through the 500 μL of 2× HBS with the use of a 1 mL serological pipette attached to a pipette aid while adding the CaCl_2_-DNA solution drop-wise. Let the mix sit at RT for 30 min. You should be able to see the development of a cloudy solution due to the formation of the precipitate.

**CRITICAL:** It is important that you add the 500 μl CaCl_2_-DNA solution to the 500 μl HBS solution (1:1 ratio) dropwise while introducing turbulence to the mix. For the successful creation of a single vector particle, copies of all 5 plasmids need to enter the nucleus simultaneously. Therefore, the solution needs to be homogenous. Constant turbulence will prevent saturation of the mix and will create smaller precipitates. Bigger precipitates will reduce the viability of the culture.

20. 2 h after medium exchange of the plates containing the CD46KO 293T cells, pipette the prepared DNA/CaPO_4_ suspension up and down multiple times and then slowly add the 1 mL precipitate to each plate. Rock back and forth to evenly distribute precipitate. Incubate cells for 16 h at 37°C in a humidified 5% CO_2_ incubator.

Day 3

21. Approximately 16 h later, carefully remove all the medium/transfectant from the cells and discard. Add 5 mL of 3% FBS-DMEM to cells and incubate for 24 h. The use of lower serum concentrations and the absence of antibiotics in the culture medium have been associated with improved lentiviral vector purity and higher viral titers after downstream processing.

Day 4

22. Collect and filter MV-LV containing supernatant from 3 x 10 cm^2^ plates through a 0.22 μm PES filter to remove cell debris. Transfer the 15 mL of purified MV-LV containing supernatant into ultracentrifuge tubes.

***Note:*** Increased purity is required for transduction of primary cells therefore viral vector should be layered onto a 5 mL of filter-sterilized 20% (w/v) sucrose/HBSS cushion.

**CRITICAL:** Perform only one harvest on day 4. Other protocols suggest refrigeration of harvested supernatant on day 4 with the addition of harvested supernatant from day 5. Direct comparisons of MV-LV titers produced from 1 harvest on day 4, two harvests on days 4 and 5, and independent harvest on day 5 showed that even though the physical titers increase with the combination of harvests from day 4 and 5, primary B cell transduction was decreased. Our findings suggest that the MV envelope may not be stable overnight at 4°C and/or that the increase of physical titers is due to the detection of virus like particles (VLPs) produced on day 5.

**CRITICAL:** Different filter pore sizes and filter membrane types allow for different viral vector recovery propensity (Nilsson, 2016). Avoid using high protein binding filters as their use will decrease recovery of MV-LV. Our observation has been that low protein binding PES membrane is the most appropriate type of filter for the purification/cell debris removal step. The use of a 0.45 μm membrane will result in higher MV-LV recovery. However, due to the low viability of human primary B cells in culture we chose to proceed with 0.22 μm PES filters for increased purity.

**CRITICAL:** Poly-L-Lysine was used to coat transfection plates to prevent detachment of producer cells from the plate. However, if CD46KO 293T cells detach and are observed in the supernatant, centrifuge the collected supernatant at 400 × *g* for 5 min at 4°C before step 22 to prevent filter clogging.

23. Next, place ultracentrifuge tubes into the SW28 Ti rotor, making sure to balance with appropriately weighted tubes. Sterile PBS can be used to balance ultracentrifuge tubes.
24. Ultracentrifuge at 19,400 rpm using an SW28 Ti rotor, or at 67,865 × *g* for any other rotor, for 2 h 20 min at 4°C. Brake should be set to slow when using the Optima^TM^ L-90K Ultracentrifuge.

**CRITICAL:** Take extra care at this step to not dislodge the pellet when removing the ultracentrifuge tubes from the rotor. Directly proceed to step 26. Our observations suggest that temperature appears to have an important effect on MV-LV titer, possibly due to MV-LV stability.

25. After collecting the MV-LV supernatant from the CD46KO 293T cell containing plates determine plasmid transfection efficiency using either fluorescence microscopy or flow cytometry. To collect cells for flow cytometry, add 1 mL of TrypLE and incubate plate at 37°C for 5 min. Add 100 μL of dissociated transfected CD46KO 293T cells in 900 μL of PBS and proceed with flow cytometry sample analysis.

***Note:*** Transfection efficiency should be ≥90% to achieve appropriate LV titer amounts to be able to achieve high transduction levels in primary B cells (Figure 1A). The relationship between transfection and transduction efficiency is not linear. A relatively small drop in transfection efficiency appears to have a disproportionately negative effect on transduction efficiency of primary B cells. Therefore, ensure high plasmid transfection efficiency to achieve high MV-LV titers for further use.

26. After ultracentrifugation is completed, carefully remove tube, decant supernatant into a 10% bleach solution and invert tubes onto a sterile paper towel inside a tissue culture hood. Leave tubes inverted for 2 min to allow extra supernatant to drip onto the paper towel.
27. Resuspend pellet in 75 μL IMDM to achieve a 150× concentration. To recover pelleted vector, pipette up and down in IMDM at least 100 times, while limiting foaming.
28. After resuspension, aliquot vector, being sure to label with MV-LV name and date of production.

***Note:*** We suggest resuspending the MV-LV in 75 μl IMDM per 3 × 10 cm^2^ plate collected (collected MV-LV volume=15 mL) to achieve significant transduction efficiencies in primary human B cells. Based on our observations, concentrated MV-LV from 1 × 10 cm^2^ transfection plate can efficiently transduce 3 × 10^5^ primary B cells seeded in 1 well of a 96 well plate. It is important to transfect multiple 10 cm^2^ plates rather than larger size flasks to ensure high transfection efficiencies of the 5 independent plasmids.

29. Aliquot 75 μL of resuspended MV-LV in cryovials placed on ice. Immediately store vector at −80°C. Titrate using either functional titer (described below) or by p24 ELISA (Takara) following the manufacturer’s protocol (https://www.takarabio.com/documents/User%20Manual/Lenti-X%20p24%20Rapid%20Titer%20Kit%20User%20Manual_022621.pdf).

**CRITICAL:** Storage temperature and temperature changes are known to affect MV-LV functionality and stability. Any higher temperature will significantly decrease functional titers. Make sure to aliquot vector to prevent multiple rounds of freeze-thaw. The vector is stable for long term storage at −80°C but a single round of freeze-thaw can decrease titers by as much as ∼50%.

### MV-LV titration

**Timing: 4 days**

Before proceeding to primary B cell transductions titrate the produced MV-LV to ensure high titer stock. The methodology below describes the steps that should be followed to accurate measure MV-LV viral titers.

30. Count and seed 3 × 10^5^ Nalm6 cells per well in a 6-well plate in a final volume of 2 mL cRPMI. 12 wells of Nalm6 cells should be seeded to include technical duplicates of the 5 serial dilutions and two mock transduction samples.
31. 18 h post seeding, thaw viral supernatants to transduce Nalm6 cells to calculate viral titers. Nalm6 was chosen as an appropriate cell line to titrate measles virus pseudotyped LV due to its permissive nature and B cell lineage phenotype. Nalm6 cells need to be approximately 60% confluent on the day of transduction.
32. Create a series of viral supernatant 1/10 serial dilutions from 10^-1^ to 10^-5^ in a final volume of 600 μL cRPMI. LVs used for titration were stored at −80°C for a minimum of 18 h before thawing to perform serial dilutions.
33. Replace old medium with 500 μL fresh cRPMI containing 8 μg/mL polybrene.

***Note:*** Polybrene is a cationic polymer that neutralizes the charge repulsion between virions and cells thereby increasing transduction efficiency.

34. Add 500 μL of each viral dilution to the 500 μL polybrene-containing cRPMI, mix by pipetting, and return plates to a 37°C, humified 5% CO_2_ incubator for 2 h.

***Note:*** Establish triplicates per each dilution in the series. Always seed two extra wells to use as mock transduced controls.

35. 2 h later add 1 mL of cRPMI and return plates to a 37°C, humified 5% CO_2_ incubator.
36. 48 h post transduction prepare cells for flow cytometry by collecting 100 μL of each cell well per condition and mix with 400 μL of PBS.

***Note:*** Titers should be calculated using values from the transduced wells in which the proportion of eGFP positive cells was between 2.5% and 25%. If the transduction efficiency is higher, it is likely that multiple vector particles could have transduced the same cell resulting in an underestimate of viral titers. Lower transduction frequencies, less than 2.5%, are unreliable. Calculate the transduction efficiency of each lentiviral vector by multiplying the number of seeded cells with the percentage of eGFP positive cells and with the vector dilution factor, divided by the final volume of culture (1 mL) per transduction well.

**Figure 2:**
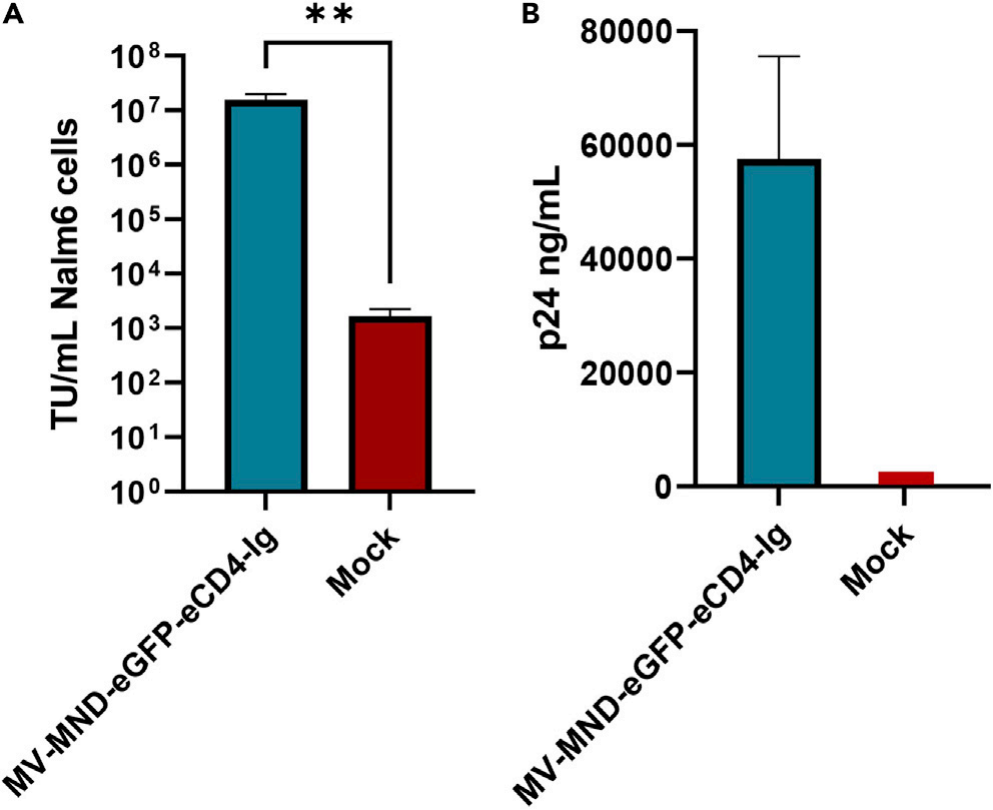
Measurement of functional and physical titers for MV-LV (A) Measurement of infectious titers for Measles virus-pseudotyped LVs (MV-LV) in Nalm6 cells. Nalm6 cells were transduced with varying dilutions of MV-LVs as described above in triplicates. Functional titers were calculated by flow cytometry of eGFP-expressing cells, using the dilution in which samples contained 2.5%–25% eGFP+ cells. Graph shows mean ± S.D. of triplicates. (B) Measurement of physical titers of MV-LV from 2 independent production preps. ELISA p24 was performed on purified and concentrated MV-LVs. Graph shows mean of mean ± S.D. of triplicates.

***Note:*** Transduction units (TU) are defined as the number of functional viral particles in the supernatant that are capable of transducing a cell and successfully expressing the transgene. A successful measles virus pseudotyped LV prep following these LV production and titration protocols is expected to generate ∼5.0 × 10^7^–5.0 × 10^8^ TU/mL (Figure 2A), while VSV-G pseudotyped LVs are generally expected to reach ∼10^9^ TU/mL. Physical titers can be measured using the Lenti-X p24 Rapid Titer ELISA Kit (Takara) following the manufacturer’s protocol (https://www.takarabio.com/documents/User%20Manual/Lenti-X%20p24%20Rapid%20Titer%20Kit%20User%20Manual_022621.pdf). Note that ELISA assays are generally characterized by higher variability compared to functional titration (Figure 2B). Also, physical titers are less informative than functional titers as they cannot exclude detection of free Gag and VLPs. A good MV-LV prep will require a 10^5^-10^6^ dilution for within range p24 measurement. If the transfer vector of interest does not contain a reporter gene, ddPCR titration is an acceptable alternative (see ddPCR for measurement of vector copy number (VCN) for protocol steps below).

### Primary B cell isolation and activation

**Timing: 2 days**

The workflow required for successful isolation and activation of human primary B cells is described below. Ensure B cell seeding in appropriate density and pre-stimulation before lentiviral mediated transduction.

**Figure 3:**
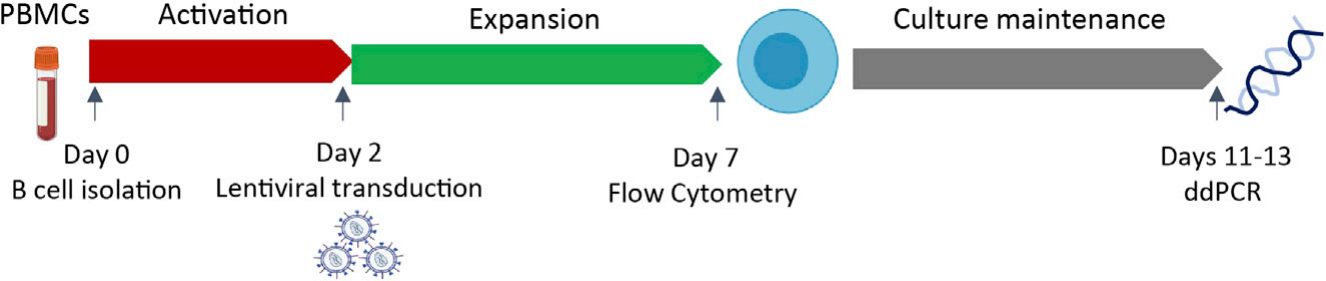
Workflow of isolation, expansion and LV transduction of primary human B cells including the analytical assays performed for assessment of transduction efficiency

***Note:*** Alternatively PBMCs can be isolated from freshly collected blood. This is expected to improve the viability of B cells which are known to be sensitive to freeze-thawing. We chose to work with pre-ordered isolated frozen PBMCs for a more reproducible and simple protocol.

37. On day 0 of the human primary B cell workflow depicted on Figure 3, thaw one vial of isolated PBMCs in a 37°C water bath. Starting PBMC concentration should be 130–150 × 10^6^ cells/mL.

38. Pipette 5 mL of PBMCs slowly and dropwise in 20 mL PBS.
39. Centrifuge PBMCs at 400 × *g* for 5 min at RT.
40. Aspirate supernatant, resuspend in 20 mL of PBS and spin down as above. Meanwhile count cells.
41. Aspirate supernatant and depending on the number of cells add up to 8.5 mL of EasySep buffer as per manufacturer’s suggestion. From this step onwards follow the protocol provided by STEMCELL’s EasySep Human B cell enrichment kit (https://cdn.stemcell.com/media/files/pis/DX20007-PIS_1_1_1.pdf?_ga=2.258265034.1508623685.1644366130-665242244.1581346724) as described below.
42. Transfer the total volume in a 14 mL polystyrene round bottom tube. Add 50 μL of enrichment cocktail per mL of sample. Leave at RT for 10 min.
43. Vortex magnetic beads for 30 s. Mix 75 μL of magnetic beads per mL of sample. Leave at RT for 5 min.
44. Add up to 5 mL of EasySep buffer.
45. Transfer tube into a magnet and incubate at RT for 5 min.
46. Invert the magnet containing the tube and pour the enriched B cell suspension into a new sterile tube.
47. Count cells and resuspend in B cell expansion media in a seeding density of 1.5 × 10^6^ cells/mL in a final volume of 3–5 mL/well of a 6 well plate.

### MV-LV transduction

**Timing: 5 days**

Steps 48–55 detail the methodology that should be followed to achieve high transduction efficiency of human primary B cells.

48. On day 2 (Figure 3) collect and pool all cells. Count cells and resuspend in IMDM in a final concentration of 1.5 × 10^6^ cells/mL.

**CRITICAL:** It is important to seed cells at the suggested seeding density as cell density is an important parameter for primary B cell viability.

49. Seed activated primary B cells at 3 × 10^5^ cells/well of a 96 well clear flat-bottom plate.
50. Add 20 μL (5.0 × 10^7^–1 × 10^8^ TU/mL) of MV-LV (Figure 3) and create technical triplicates for each transfer vector or experimental condition of interest.
51. Spinoculate for 30 min at 400 × *g* at RT and leave in a 37°C, humified 5% CO_2_ incubator for 6 h.
52. 6 h later centrifuge the 96 well plate at 400 × *g* for 5 min at RT.
53. Remove 100 μL of media and replace with 100 μL of fresh 2× B cell expansion medium (all additives included in the B cell expansion IMDM medium should be at a 2× concentration) to achieve a final volume of 220 μL 1× B cell expansion medium. Return plate to a 37°C, humified 5% CO_2_ incubator.
54. On day 3 (Figure 3) transfer cultures from each well of the 96 well plate to a 48 well flat bottom plate and add 200 μL 1× B cell expansion medium to each well for a final volume of 420 μL.
55. On day 5 (Figure 3) transfer cultures from each well of the 48 well plate to a 24 well flat bottom plate and add 400 μL 1× B cell expansion medium for a final volume of 820 μL.

### Flow cytometry, surface and intracellular staining

**Timing: 8–9 h**

Flow cytometry can be used to determine transduction efficiency as well as the potential effect of lentiviral mediated transduction on B cell phenotype. The following protocol describes the staining process for the most important B cell markers. Transduction efficiency is assessed using the eGFP marker incorporated in the transfer vector.

56. On day 7 (Figure 3) count and transfer 2 × 10^5^ B cells per condition to a 96 well round bottom plate. Add 100 μL of PBS and centrifuge for 5 min at 400 × *g*.
57. Decant supernatant. Add 200 μL of PBS and repeat spin step as above.
58. Meanwhile prepare an extracellular staining master mix that contains AF350 LIVE/DEAD^TM^ fixable violet dead cell stain (dilution 1:1000), Brilliant Violet 605™ anti-human CD19 antibody (dilution 1:200) and Brilliant Violet 785™ anti-human CD3 antibody (dilution 1:200) in PBS.
59. Decant supernatant. Add 50 μL of extracellular staining master mix. Pipette up and down multiple times. Cover plate and leave it at 4°C for 30 min in the dark.
60. Once the extracellular staining step is complete, add 200 μL of PBS and repeat spinning step as above.
61. Decant supernatant and add 100 μL of Cytofix/Cytoperm™ fixation and permeabilization solution. Cover plate and leave it at RT for 20 min in the dark.

**CRITICAL:** It is very important to thoroughly mix the cells in the Cytofix/Cytoperm™ solution using gentle pipetting for efficient intracellular staining. Do not exceed incubation time for more than 20 min.

**Figure 4:**
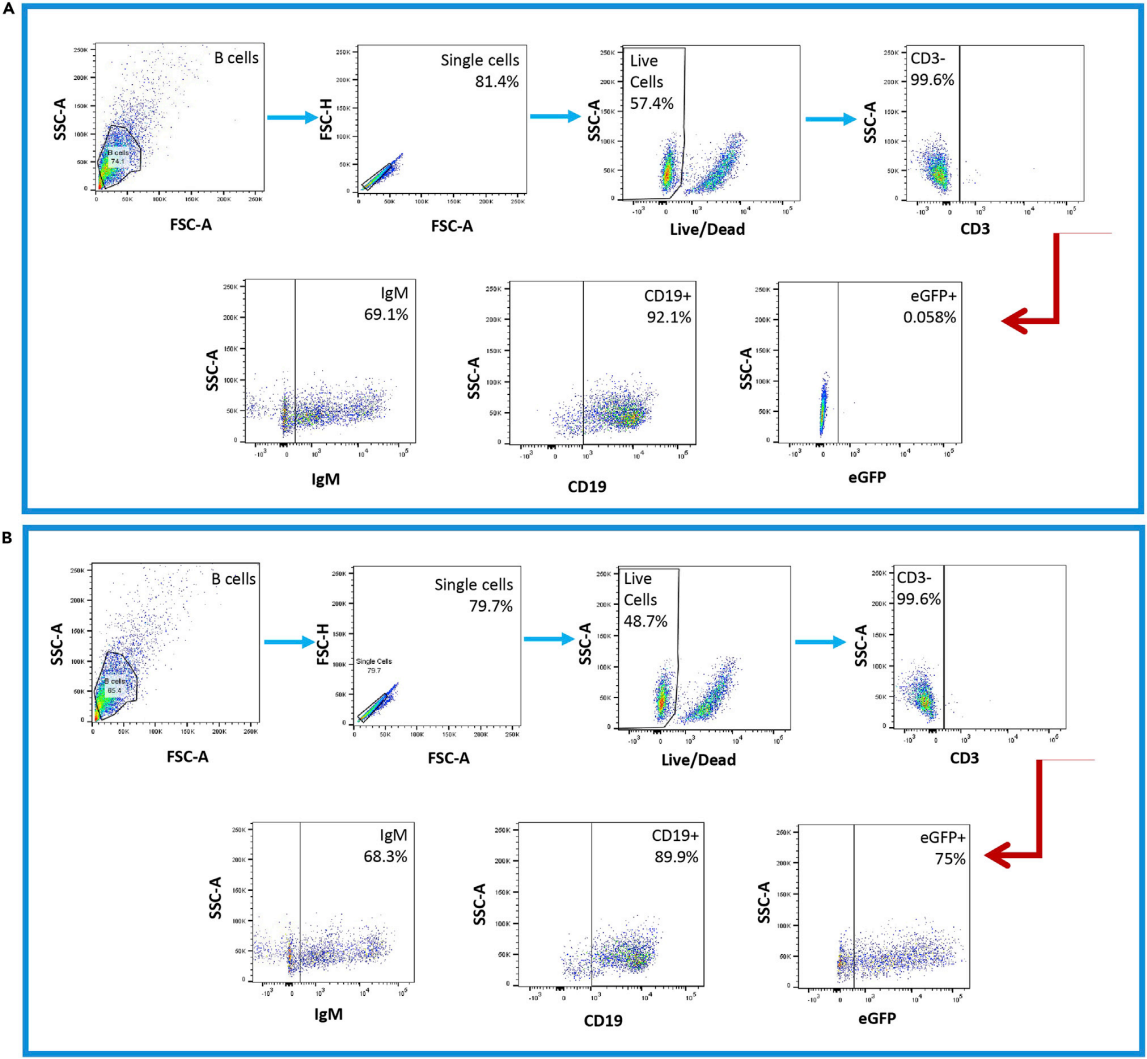
Gating strategy for effective flow cytometric analysis of human primary B cells. Gating strategy for effective flow cytometric analysis of (A) Mock and (B) Transduced human primary B cells. Doublets and dead cells were excluded by FSC, SSC, and AF350 LIVE/DEAD^TM^ fixable violet dead cell stain. T cells were excluded by selecting the CD3- population. Transduction efficiency was assessed by measuring the eGFP+ population. B cell phenotypic analysis was performed by CD19 marker staining and immunoglobulin expression was assessed with IgM staining.

62. Add 200 μL of Perm/Wash™ Buffer and spin as above.
63. Decant supernatant and repeat step 62.
64. Decant supernatant and add 100 μL of Cytoperm™ Permeabilization Buffer Plus. Cover plate and leave it at 4°C for 10 min in the dark.
65. Add 200 μL of Perm/Wash™ Buffer and spin as above.
66. Decant supernatant and repeat step 65.
67. Decant supernatant and add 100 μL of Cytofix/Cytoperm™ Fixation and permeabilization solution. Cover plate and leave it at RT for 5 min in the dark. Even though most protocols use a single fixation/permeabilization step, we have noticed increased IgM detection by introducing this extra step.
68. Add 200 μL of Perm/Wash™ Buffer and spin as above.
69. Decant supernatant and repeat step 68.
70. Meanwhile prepare intracellular staining master mix that contains PE/Cyanine7 anti-human IgM antibody (dilution 1:200) in Perm/Wash™.
71. Decant supernatant. Add 50 μL of intracellular staining master mix. Pipette up and down multiple times. Cover plate and leave it at 4°C for 30 min in the dark.
72. Meanwhile prepare compensation beads. Mix 3 drops of positive beads with 3 drops of negative beads and 1 μL of each antibody used respective tube. Mix well and place rack at 4°C for 30 min in the dark.
73. Once the intracellular staining step is complete, add 200 μL of Perm/Wash™ and repeat spinning step as above.
74. Decant supernatant and repeat step 73.
75. Decant supernatant and resuspend pelleted stained cells in 250 μL PBS. Proceed to flow cytometry. Flow cytometry was performed with a BD LSRII machine equipped with UV, violet, blue, and red lasers using the following reagents and settings. Flow cytometry gating strategy is presented in Figure 4. Voltages are included in the table below for convenience if using the BD LSRII flow cytometer. However, voltage optimization should always be performed on the user flow cytometer in the beginning of any acquisition to ensure appropriate compensation.

**Table:** 

Flow cytometry reagent	Dilution	Voltages
AF350 LIVE/DEAD^TM^ fixable violet dead cell stain	1:1000	250
Brilliant Violet 605™ anti-human CD19 antibody	1:200	440
Brilliant Violet 785™ anti-human CD3 antibody	1:200	400
PE/Cyanine7 anti-human IgM antibody	1:200	400
Forward scatter (FSC)	N/A	350
Side scatter (SSC)	N/A	260
eGFP	N/A	420

***Note:*** Measurement of transduction efficiency following our protocol using the bicistronic eGFP-eCD4-Ig vector under the constitutive MND promoter was determined to be ∼75% on average. Long inserts and complex cassettes have been associated with reduced titers due to incomplete vector RNA and lowered virion production (Han et al., 2021). Hence, we had chosen to work with a bicistronic vector that contains both eGFP and a therapeutic transgene with immunoglobulin containing properties (Gardner et al., 2015), also associated with reduced titers, as a model vector for protocol optimization. Therefore, even though a certain level of variation is to be expected, this protocol has taken into account the common LV packaging limitations.

***Note:*** Transduction efficiency and viability are measured 5 days post MV-LV mediated transductions (Figure 3). Viability of the genetically modified B cells is expected to be between 45%–50%, while viability of Mock B cells has been observed to be 55%–58% (Figure 4). The purity of the MV-LV prep significantly affects B cell viability. The remaining B cell culture can be maintained in B cell expansion medium for approximately one additional week or used for differentiation and transplantation studies as described before (Hung et al., 2018). By the end of day 13 of human primary B cell culturing (Figure 3), B cell viability is expected to drop to 10%, therefore the maximum duration of B cell culture, including all steps of activation, expansion, transduction and further culturing, as described in Figure 3, is approximately two weeks.

### Genomic DNA (gDNA) extraction from human B cells

**Timing: 1.5 h**

76. Collect and centrifuge all activated human primary B cells for 5 min at 300 × *g* at RT. VCN should be measured at 7–11 days post transduction to ensure degradation of non-integrated provirus (Figure 3).

**CRITICAL:** It is important to collect all primary B cells for gDNA extraction. As mentioned above, one of the limitations of the *in vitro* culture of human primary B cells is the high death rate. Extraction of gDNA from a small number of B cells of which approximately 50% are expected to be dead will lead to an overestimation of VCN due to DNA degradation. Therefore, it is of paramount importance to use the maximum number of B cells possible.

77. Resuspend pellet in 200 μL PBS and proceed to gDNA extraction using the DNeasy Blood and Tissue kit (Qiagen).
78. Add 20 μL proteinase K and 200 μL of Buffer AL. Mix thoroughly by vortexing and incubate samples at 56°C for 10 min for efficient cell lysis.
79. Add 200 μL pure ethanol and mix the sample thoroughly by vortexing. Pipet the sample into a DNeasy Mini spin column placed in a 2 mL collection tube and centrifuge at ≥6,000 × *g* for 1 min at RT. Discard the flow-through and return the spin column to the same collection tube.
80. Add 500 μL of Buffer AW1 in the spin column and centrifuge as above.
81. Add 500 μL Buffer AW2 in the spin column and centrifuge for 3 min at 20,000 × *g* at RT. Discard the collection tube.
82. Transfer the spin column to a new 1.5 mL microcentrifuge tube. Elute the DNA by adding 50 μL of water to the center of the spin column membrane. Incubate for 5 min at RT. Centrifuge for 1 min at ≥ 6,000 × *g* at RT and proceed to nanodrop.

**CRITICAL:** Elute DNA in 50 μl of water. Manufacturer’s protocol suggests a 200 μl final volume, however due to the small number of live B cells with intact chromosomal DNA, addition of a a high elution volume can lead to diluted gDNA concentrations that will not suffice for the subsequent digital droplet PCR (ddPCR) reaction. Elution of DNA in a volume smaller than 50 μl will be inadequate for sufficient membrane hydration and will also lead to small gDNA concentrations. Following this protocol you should expect to get a gDNA concentration of 10–50 ng/ml. Do not proceed to the next step unless can see a distinct 260 nm peak of ≥10 ng/mL gDNA.

### ddPCR for measurement of vector copy number (VCN)

**Timing: 2 days**

An alternative method of assessing transduction efficiency, particularly in the absence of a marker in the transfer vector, is the measurement of integrated vector copy number. This protocol can be used in conjunction with, or instead of, flow cytometry.

83. Sample preparation
  a. Dilute primers and probes at 100 μM. Then, prepare a 20× concentration stock for primers and probes by mixing 59 μL of water with 5 μL of probe, 18 μL of forward primer and 18 μL reverse primer for each target. Targets used here include the PBS of the HIV-1 leader, present in the integrated provirus, and the MKL2 gene, known as myocardin transcription factor B, present in each chromosome allele as a single copy.
  b. Add 25 ng of extracted genomic DNA to a 25 μL PCR reaction mix containing 2× ddPCR Supermix for probes, a 20× primer/probe set (p+p) for the HIV-1 target sequences and a 20× p+p set for MKL2 reference gene. The final concentration of each primer should be 0.9 μM and the final concentration for each probe 0.25 μM. Prepare this master mix as presented in the table below.

**Table:** 

Reagent	1xrn (25 μL)	2xrn 50 μL
2× ddPCR SuperMix	12.5 μL	25 μL
20× p+p HIV	1.25 μL	2.5 μL
20× p+p MKL2	1.25 μL	2.5 μL
gDNA	25 ng	50 ng
Ultra Pure water	up to 25 μL	up to 50 μL

**CRITICAL:** 25–100 ng is the observed optimal range of genomic DNA for a successful ddPCR reaction.

84. Droplet generation
  a. Place an 8 channel disposable droplet generator cartridge into a cartridge holder. Add 20 μL of the reaction sample into each cartridge well and then load 70 μL of droplet generation oil into each sample. Attach the gasket to the cartridge holder and place the set in the droplet generator. Repeat per sample to create technical duplicates. Always load the sample first followed by addition of the droplet generation oil to prevent blockage of the cartridge channel microfluidics.
  b. Remove the cartridge holder from the droplet generator and discard the gasket. Carefully transfer 40 μL of each droplet emulsion to a 96-well propylene plate with a multichannel pipette.
  c. Heat-seal the plate with foil using the PX1 PCR plate sealer, and place the plate in a Bio-Rad T100 Thermal Cycler. PCR was performed using the following thermal cycling conditions:

**Table:** 

Steps	Temperature	Time	Cycles
Initial Denaturation	95°C	10 min	1
Denaturation	94°C	30 s	39 cycles
Annealing	60°C	1 min	
Final extension	98°C	10 min	1
Hold	12°C	forever

**Figure 5:**
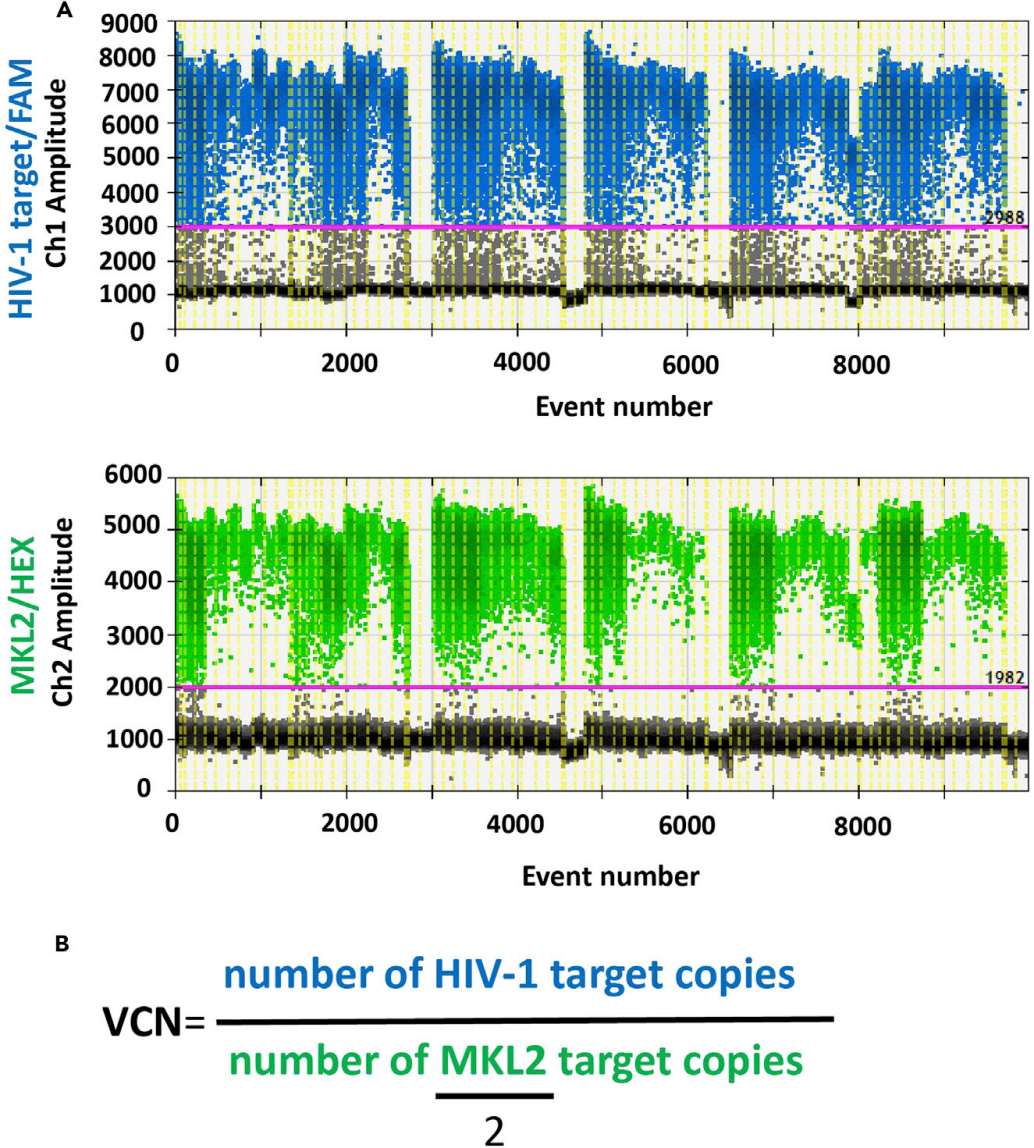
VCN analysis with ddPCR (A) 1-D fluorescence amplitude plots of the FAM-labeled HIV target assay (top) and the HEX-labeled reference gene (bottom). Plots show all accepted droplets of the plate wells in both Channel 1 (FAM/HIV) and Channel 2 (HEX/MKL2). Established threshold differentiates between negative droplets, displayed in gray, and positive droplets in Channel 1, displayed in blue, and positive droplets in Channel 2, displayed in green. (B) Equation used to calculate VCN.
  c. Calculate VCN by using the following equation: VCN=[HIV]/([MKL2]/2). The concentration of MKL2 is divided by 2, as there are two copies of the MKL2 in each cell. The VCN is then calculated by dividing the total concentration of HIV to MKL2 to measure the copies of integrated provirus per cell (Figure 5B).

***Note:*** To our observation, a transduction efficiency of 75% was associated with a VCN of 2–3, while a transduction efficiency of 50% was associated with a VCN of 1–2. VCN cannot provide information with regards to the function or expression of the transgene, therefore in the absence of a reporter marker we suggest the additional performance of analytical assays such as RT-qPCR, ELISA and, or, Western blots.

85. Droplet reading
  a. After the reaction is complete, transfer the 96-well plate to a QX100 Droplet Reader for data acquisition.
  b. Load the Quantasoft software, click ‘‘Setup’’ and then select ‘‘VCN’’ as the type of experiment. Assign HIV-1-FAM as Channel 1-Unknown and MKL2-HEX as Channel 2-Reference.
  c. Save your experimental set up and press ‘‘Run’’ to start the data acquisition process. The run indicator light flashes green to indicate that the droplet reading is in progress.
86. Data analysis
  a. When the droplet reading is complete, all four indicator lights will turn green. Open the door and remove the plate holder from the unit. Click ‘‘Analyze’’ to proceed to data analysis.
  b. Set the gates (in 1D Amplitude) by identifying the negative and positive fluorescence signals in the positive control sample reactions (Figure 5A). Export your data.

## Expected outcomes

Our protocol sequentially details the steps for efficient genome modification of human primary B cells through measles virus gp pseudotyped lentiviral vector mediated transduction. We provide an optimized working protocol for the production of high titer MV-LV. The potential of B cell based therapeutics can be now further explored with the use of our detailed protocol. We anticipate that the presented production system can be used to consistently achieve transduction efficiency of primary B cells up to 75%, including with the use of complex and long cassettes. To achieve this, each major step of this protocol should be executed as described with the following expected outcomes:

Step 1: Plasmid preparation: plasmid yield should be ≥2 μg/mL.

Steps 2–3: Measles virus pseudotyped lentiviral vector production and titration: MV-LV viral titers should be ≥5.0 × 10^7^ TU/mL

Step 4: Isolation and expansion of human primary B cells: isolation and expansion of primary B cells should allow for a seeding density of 1.5 × 10^6^/well in both seeding steps (for expansion and transduction).

Step 5: Transduction: should consistently be ∼75% with the use of sequences of similar length and complexity. Transduction efficiency will vary depending on transfer vector length and complexity of transgene cassette.

Step 6: Measurement of VCN: a transduction efficiency of 75% is expected to fall within the range of 2–3 integrated vector copy numbers per cell.

However, at each of these steps, it is possible that the obtained outcome may differ from the desired outcome due to technical difficulties. Therefore, in the troubleshooting section, we outline potential limitations and provide solutions to ensure that each of the steps leading to efficient transduction of human primary B cells is feasible.

## Limitations

B cells are generally refractory to VSV-G LV transduction. We have successfully used the described MV-LV protocol to produce high titer MV-LV which consistently transduces primary human B cells up to 75%. Historically, published methodologies generated low titer MV-LV. Therefore, big amounts of LVs have been required to efficiently transduce human primary B cells. To increase viral titers we used: i) a CD46KO 293T cell line that prevents the previously observed formation of MV-mediated syncytia during MV production (Ozog et al., 2019), ii) modified the transfection methodology and by iii) establishing simple but efficient downstream processing of the produced MV-LV. Despite the overall improvement of MV-LV viral titers and primary human B cell transduction efficiencies, large amounts of MV-LV are still required for efficient transduction; to our observation, concentrated MV-LV supernatant from a 1 × 10 cm^2^ transfection plate is required to efficiently transduce 3 × 10^5^ primary B cells seeded in 1 well of a 96 well-plate. This suggests that a large number of 10 cm^2^ transfection plates will be required for *in vivo* studies. Larger scale production of MV-LV in flasks or tissue factories remains challenging to this day. This is due to the limitation posed by the large number of plasmids that have to be simultaneously co-transfected and the instability of the MV envelope that prevents multiple serial harvests. In our experience, an increase in the scale of production was associated with lower transfection of plasmids in the CD46KO 293T cell line as well as decreased MV-LV transduction efficiencies. In addition, determining MV-LV titer using permissive cell lines, such as Nalm6 and 293T cells, does not directly translate to primary human B cells. These cell lines underestimate functional MV-LV titer by a factor or 2 and 2.5 respectively. Therefore, as a general rule we suggest the use of a MV-LV prep for transduction experiments only if the TU/mL are determined to be ≥ 5.0 × 10^7^ in Nalm6 cells. Finally, despite improvements in the *in vitro* culturing of primary B cells (Hung et al., 2018), less than 50% of transduced primary B cells are viable 5 days post transduction, making further *in vivo* or *in vitro* studies challenging at this time.

## Troubleshooting

### Problem 1

Low MV-LV viral titers.

### Potential solution

To achieve high MV-LV titers, transfection efficiency of the CD46KO cell line should be above 90%. To reach such high transfection efficiency with the use of 5 independent plasmids, you have to first ensure that you have produced high-quality transfection grade plasmids. CD46KO 293T cells need to be at 90% confluency on the day of plasmid transfection; cell confluency variations will result in lower titers. Additionally, the transfection mix needs to be homogenous to ensure generation of small precipitates and equal distribution of all plasmids; therefore, you have to be constantly introducing air bubbles while creating your transfection mix using HBS buffer pH 7. Repeat MV-LV production (steps 13–29) ensuring high transfection efficiency.

### Problem 2

High physical titers but low viral titers/transduction efficiency.

### Potential solution

High physical but low functional titers can mean two things: i) Gag polyprotein is known to be able to bud independently from the producer cell line. A non-homogenous transfection mix can lead to high expression of the packaging constructs that will result into independent Gag budding, rather than Gag assembly at the genomic RNA (gRNA) nucleation site in each transfected cell. In the absence of HIV-1 gRNA, Gag proteins can still assemble and release efficiently but the resulting VLPs are non-infectious (Rulli et al., 2007). Therefore, the high physical titers measured are likely due to the presence of Gag associated VLPs that lack gRNA. Repeat MV-LV production (steps 13–29) ensuring homogeneity of transfection mix. ii) MV-LV degradation. Measles virus envelope is less stable than VSV-G (Dautzenberg et al., 2021), therefore any alterations in downstream processing and storage can have a negative effect on the stability of the viral envelope and its infectivity. Ensure proper processing and storage of MV-LV (steps 22–29). Aliquot MV-LV to avoid multiple rounds of freeze-thawing.

### Problem 3

Low B cell viability for flow cytometry analysis

### Potential solution

It is expected that less than 50% of transduced human primary B cells will be viable 5 days post B cell transduction. Despite improvements in *in vitro* human primary B cell culture (Hung et al., 2018), B cell expansion and viability remain challenging. Ensure that the provided cryo-preserved PBMCs are of high viability before proceeding to B cell isolation. The seeding density of B cells should be 1.5 × 10^6^/well; any variations in cell density will result in variations in cell proximity and nutrient availability that will subsequently negatively impact the viability of the culture (steps 37–47). Follow the expansion steps by doubling the culture with fresh B cell expansion medium on the suggested time points (steps 54–55). If these steps don’t work, ensure proper MV-LV purification by following the filtering and ultracentrifugation steps outlined in this protocol to reduce MV-LV associated toxicity (steps 22–29). Additionally, collect more cells for flow cytometry (step 56).

### Problem 4

No gDNA or ddPCR signal.

### Potential solution

As alluded to above, expansion of human primary B cells *in vitro* remains a challenge. Collect as many B cells as possible for gDNA extraction (step 76) and make sure to elute gDNA in 50 μL of distilled water (step 82). The longer the duration of the B cell culture (Figure 3) the higher the death rate observed. Dead cells contain degraded gDNA that can interfere with the accuracy of the TaqMan assay. Perform ddPCR 6 days post transduction for maximum intact gDNA yield.

### Problem 5

Very high measured VCN with low transgene expression signal measured by alternative analytical assays

### Potential solution

The use of flow cytometry to measure transduction efficiency with the use of a fluorescent marker is a very robust and reliable method of evaluating the levels of gene delivery. On the contrary, PCR amplification for the assessment of integrated provirus is a more demanding assay. Despite technical challenges, in the absence of a fluorescent marker, VCN assessment is an accurate alternative for the measurement of transduction efficiency. If there is a lack of correlation between the two assays (or a lack of correlation between ddPCR VCN data and any other analytical assay employed for the assessment of transgene expression), ensure that you culture the primary B cells up to 11 days post transduction to ensure that you are not detecting any non-integrated reverse transcription products. Unlike flow cytometry (steps 56-75) that is commonly performed only 3–5 days post transduction, assessment of VCN with ddPCR (steps 76-83) (or any PCR amplification methodology) should be performed 7–11 days post transduction (depending on human primary B cell viability) to prevent overestimation of VCN.

## Resource availability

### Lead contact

Further information and requests for resources and reagents should be directed to and will be fulfilled by the lead contact, Bruce Torbett (betorbet@uw.edu).

### Materials availability

Currently we are combining the two measles envelope sequences into a single plasmid to make pseudotype vector production easier. We intend to deposit this plasmid at Addgene. In the interim, we ask that interested groups contact us for the plasmids. The Scripps Research Institute, La Jolla, CA, USA, is in the process of depositing the CD46KO 293T cell line we developed at the National Gene Vector Biorepository (https://www.ngvbcc.org/Home.action).

## Data Availability

This study did not generate or analyze any dataset or code.
